# Bioelectrical Impedance Phase Angle as an Indicator of Malnutrition in Hospitalized Children with Diagnosed Inflammatory Bowel Diseases—A Case Control Study

**DOI:** 10.3390/nu10040499

**Published:** 2018-04-17

**Authors:** Paweł Więch, Mariusz Dąbrowski, Dariusz Bazaliński, Izabela Sałacińska, Bartosz Korczowski, Monika Binkowska-Bury

**Affiliations:** 1 Institute of Nursing and Health Sciences, Faculty of Medicine, University of Rzeszów, 35959 Rzeszów, Poland; mariusz.dabrowski58@gmail.com (M.D.); darek.bazalinski@wp.pl (D.B.); izabela.salacinska@wp.pl (I.S.); mbinkowskabury@gmail.com (M.B.-B.); 2 Diabetic Outpatient Clinic, Medical Center “Beta-Med”, 35073 Rzeszów, Poland; 3 Pediatric Department, Clinical Provincial Hospital No. 2 in Rzeszów, Faculty of Medicine, University of Rzeszów, 35301 Rzeszów, Poland; korczowski@op.pl

**Keywords:** phase angle, bioelectrical impedance, inflammatory bowel diseases, malnutrition, children

## Abstract

The phase angle (PhA) seems to be a reliable screening tool for the identification of malnutrition risk in hospitalized children with inflammatory bowel disease (IBD). The aim of the present study was to assess the body composition and nutritional status of hospitalized children and adolescents with IBD by using bioelectrical impedance analysis (BIA) with phase angle (PhA) calculation, which has not been evaluated in hospitalized children with IBD yet. A total of 59 children and adolescents aged 4–18 years, with IBD: 34 ulcerative colitis (UC) and 25 Crohn’s disease (CD) were included in the study. The control group consisted of healthy children and adolescents, strictly matched for gender and age in a 1:1 case-control manner. In both groups, BIA was performed and PhA was calculated. IBD patients had significantly lower PhA (UC: 5.34 ± 1.34 vs. 5.96 ± 0.76, *p* = 0.040; CD: 5.16 ± 1.18 vs. 5.90 ± 0.62, *p* = 0.009) compared to the control subjects. Significant changes in selected body composition parameters were observed particularly in CD, especially in fat free mass components. Lower phase angle score together with lower body composition parameters and selected nutrition indicators in children and adolescents with IBD demonstrate their worse nutritional and functional status compared to healthy subjects.

## 1. Introduction

Bioelectrical impedance analysis (BIA) has been used since the late 1980s as a portable, easy-to-use, inexpensive, non-invasive and safe technique for assessing body composition [1,2,3]. The detection of nutritional status in children with different clinical conditions requires the measurement of both fat mass (FM) and fat free mass (FFM). Body cell mass (BCM) is the metabolically active component of FFM and reflects the functional status of the cellular component of the body [4]. BIA-derived phase angle (PhA) is a variable obtained from the relationship between Resistance (R) and Reactance (Xc) (R/Xc × 180°/π) and seems to be a new promising indicator of nutritional status [2]. Its biological meaning is not completely understood, although it is interpreted as an indicator of membrane integrity and water distribution between intracellular and extracellular compartments [5].

PhA score correlates with functional status. PhA is an important predictor of mortality [6,7,8], malnutrition in cancer [8,9,10,11], liver cirrhosis [12], diabetes [13,14], psoriasis [15], in chronic kidney disease treated with hemodialysis [16] and in heart failure [17]. Hence, PhA can be regarded as a reliable prognostic marker and should be considered as a screening tool for the identification of patients at risk of impaired nutritional and functional status [2,18]. Normative values and cut-off points for PhA score in healthy, both pediatric and adult populations, were assessed in two large cross-sectional studies [19,20]. PhA is strongly recommended by the European Society for Clinical Nutrition and Metabolism (ESPEN) as a prognostic nutritional measure [4,21].

In children with inflammatory bowel disease (IBD), the extent of tissue loss with the disease, and its accruement with the treatment is unclear [22]. Malnutrition is a common challenge in that group of patients, especially with active Crohn’s disease (CD) [22,23,24]. In the study by Werkstetter et al., the phase angle has been assessed in pediatric IBD patients during clinical remission. In this study a low phase angle score and a reduced lean body mass was revealed [25]. It was also confirmed in our study [26]. 

The primary objective of our study was to assess PhA score in children and adolescents with IBD compared to healthy controls. To the best our knowledge, it is the first study conducted in hospitalized pediatric IBD patients with different stages of disease activity. While searching EMBASE, Web of Science, PubMed and Scopus databases, we found only 1 full-text paper assessing PhA in pediatric IBD patients in remission or with only mild disease activity [25]. The secondary objective was to analyze body composition in this group of patients.

## 2. Materials and Methods 

### 2.1. Ethics

The study was approved by the institutional Bioethics Committee at the University of Rzeszów (Resolution No. 5/02/2012) and by all appropriate administrative bodies. The study was conducted in accordance with ethical standards laid down in an appropriate version of the Declaration of Helsinki and in Polish national regulations. The study was conducted according to the STROBE criteria and registered www.researchregistry.com (IUN research registry researchregistry3834).

### 2.2. Subjects

The study group consisted of 59 children and adolescents (25 girls, 42.3%) with non-specific IBD (34 children with UC, and 25 children with CD) hospitalized in the Clinical Department of Pediatrics with the Pediatric Neurology Unit at the Clinical Regional Hospital No. 2 in Rzeszow. Patients with both newly diagnosed IBD as well as subjects at different stages of treatment were included. 

According to the CD Registry in Poland based on data from 95 centers, there are currently 6112 patients suffering from CD, both adults and children. In the Podkarpackie Province, where our study was conducted, 28 children with CD are currently registered. There is no nationwide register of patients with UC in Poland. However, analysis of the Epidemiological and Social Report on IBD for the year 2016, gives the information that in pre-school age UC is the most frequently diagnosed inflammatory bowel disease, while in the teenagers, CD is roughly three times more prevalent than UC. Taking into account these data, we can assume that almost all pediatric patients with CD and vast majority of children and adolescents with UC from Podkarpackie Region were included in our study.

The inclusion criteria were as follows: diagnosed non-specific IBD; age 4 to 18 years, no other autoimmune or chronic disease having impact on height, weight or nutritional status and written informed consent signed by parents or legal guardians, and also by adolescents aged over 16. 

**Figure 1:**
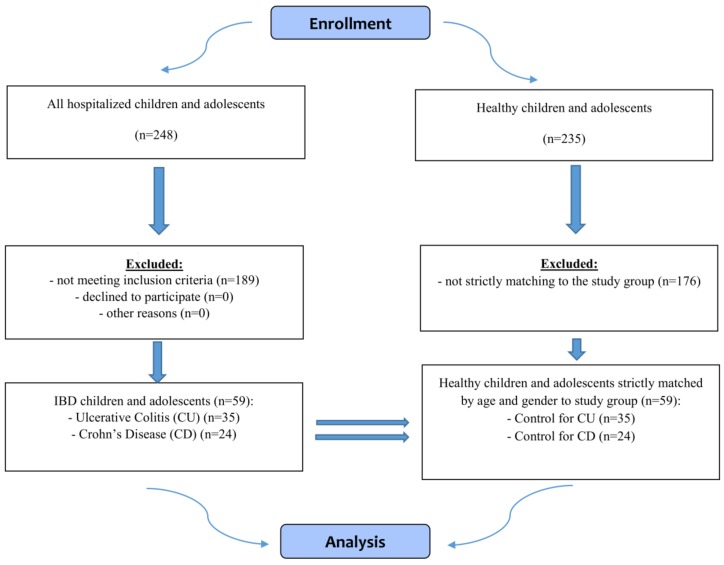
Flow chart demonstrating study participants selection.

The control group consisted of the same number of children and adolescents selected from primary, junior high and high schools from urban and rural areas. The inclusion criteria for this group were the same with the exception of non-specific IBD. The IBD and healthy subjects were strictly matched for gender and age (the nearest possible date of birth) in a 1:1 case-control manner. The flow chart demonstrating selection of the study and control groups is presented in Figure 1, while characteristics of all groups is presented in Table 1.

In the CD group, at the time of BIA measurement, 12 children (48.0%), 8 boys and 4 girls were using immunosuppressants; 5-ASA derivatives were used in 3 children (12.0%), 2 boys and 1 girl. Ten children (40.0%) (7 boys and 3 girls) did not take any medication at the time of the BIA measurement (newly diagnosed children). In the UC group 9 children (26.5%), 3 boys and 6 girls were using immunosuppressants. Glycocorticoids were used by 3 children (8.8%), 2 boys and 1 girl. 6 children (17.6%), 3 boys and 3 girls were treated with 5-ASA derivatives. 16 newly diagnosed children (47.1%), 9 boys and 7 girls did not take any drugs.

### 2.3. Assessments

In all children and adolescents weight and height were measured and BMI was calculated. Then BIA was performed using AKERN BIA-101 analyzer (Akern SRL, Pontassieve, Florence, Italy) to assess the body composition and nutritional status. The measurements were conducted between 7:00 and 12:00, in a fasting state, in supine position, with abducted upper (30°) and lower (45°) extremities, after at least 5 min of rest. A tetrapolar system with a contralateral mode was used. Amplitude of measured current was 800 μA, sinusoidal, 50 kHz. To ensure reliability and repeatability of the results obtained, two measurements, one after another, were performed. Disposable electrodes were placed on the dorsal surface of the right upper (over the wrist) and right lower limb (on the ankle). The results were analyzed with a specialized software (Bodygram1_31 by AKERN, Pontassieve, Florence, Italy).

BIA analysis included: fat mass (FM), fat free mass (FFM), muscle mass (MM) (kg and %), total body water (TBW), intra- and extra-cellular water (ICW and ECW) (liters and %), body cell mass (BCM) (kg and %) and body cell mass index (BCMI), and, upon resistance and reactance results, phase angle was calculated. In addition, fat mass index (FMI) and fat free mass index (FFMI) were calculated. 

### 2.4. Statistical Analysis

The statistical analysis was performed using the Statistical Software for the Social Sciences SPSS Statistics 20 (IBM Software Group, San Francisco, CA, USA). Normality of distribution was assessed with the Kolmogorov-Smirnov and Shapiro–Wilk tests. The continuous data are presented as mean ±SD (standard deviation). Differences between groups were analyzed with the two-tail student’s *t*-test for independent variables in case of parametric distribution, while Mann–Whitney U Test was used in case on non-parametric distribution. The categorical data were compared using χ^2^ test. A P value below 0.05 was considered statistically significant. The statistical power of our study was 0.88, which is above the lowest recommended power of 0.8.

## 3. Results

Body mass index was not significantly different between patients with UC and healthy controls, while the patients with CD had significantly lower BMI. This difference appeared to be significant only among boys and not among girls (Table 1). 

Phase angle score in the patients with IBD was significantly lower compared to the healthy subjects and it was more pronounced in the patients with CD (Table 2). 

Body composition parameters were not significantly different between the UC and control groups. In the CD patients, fat free mass, fat free mass index, muscle mass, total body water, body cell mass and body cell mass index were significantly different compared to the control group. However, variables measured as percent of mass or volume appeared to be different only with regards to body cell mass (Table 2). 

In the analysis according to gender, phase angle score was significantly lower in the girls with UC compared to the healthy subjects. They had also lower BCM%. PhA and body composition parameters in the boys with UC were not significantly different in comparison with the healthy boys (Table 3). 

Inversely, in the group with CD significant differences between the study and control groups were observed only among boys. Phase angle, muscle mass, body cell mass and total body water were significantly lower in the boys with CD compared to the healthy subjects. Among girls, no significant differences were found (Table 4). 

We performed additional analysis of phase angle scores in relation to reference values for healthy population developed by Bosy-Westphal et al. in the study conducted in Germany [20]. One fifth of all the IBD population had phase angle below the 5th percentile and compared to control groups differences were significant both for UC and CD patients. In the analysis of fat mass, fat free mass and their indices (fat mass index and fat free mass index) we observed a significantly higher proportion of participants with FFMI below 9th or 2nd percentile among children and adolescents with CD compared to healthy children using reference values for healthy population obtained by Wells et al. [27,28] (Table 5).

## 4. Discussion

The primary objective of our study was to assess the body composition and nutritional status of hospitalized children and adolescents with IBD by using bioelectrical impedance analysis with phase angle calculation in comparison to healthy subjects, which has not been studied yet. BIA-derived phase angle score consists of the resistance and reactance values and reflects nutritional and functional status. Precisely, PhA in ranges between 5° and 7° indicates higher cellularity cell membrane integrity (20), and it is also one of the best indicators of cell membrane function [29]. In the present case control study, we observed significantly lower PhA in the children and adolescents with IBD compared to the healthy controls. Also, several body composition parameters were significantly different between the IBD and control groups. Obviously, these parameters change in the course of the disease. Previous studies in pediatric IBD patients have reported a lower fat free mass in children with Crohn’s disease compared to healthy controls [30,31]. Furthermore, the study by Sylvester et al. demonstrated persistent deficits of lean mass despite implemented treatment [32]. The long-term consequences of this status are not clear, although a reduced muscle mass may be associated with less physical strain on bones and, therefore, reduced bone growth as well as predisposing to osteopenia and osteoporosis [33]. Thus, growth and nutritional status in pediatric patients with IBD should be considered in terms of body composition rather than a simple anthropometric change [34]. In their cross-sectional study, Pileggi et al. demonstrated that PhA is a sensitive method for identifying malnutrition risk at hospital admission and monitoring nutritional status of children during hospitalization [35]. 

The results of the present study showed that selected body composition parameters (fat mass, fat free mass, muscle mass, total, intracellular and extracellular body water and body cell mass) and nutritional indicators (fat mass index, fat free mass index, body cell mass index and phase angle) are lower in children and adolescents with IBD than in healthy controls. The significantly lower BIA parameters and nutritional indicators were seen predominantly in children with Crohn’s disease. In our study, PhA score was significantly lower in patients with both UC (*P* = 0.040) and CD (*P* = 0.009) groups. Wiskin et al. observed nutritional deficits in IBD children, particularly in the CD group. In these children recruited from the regional pediatric gastroenterology service, fat free mass was related to disease activity regardless of changes in weight [22]. Also, in a German study conducted in thirty-nine children and adolescents with IBD (27 Crohn’s disease, 12 ulcerative colitis), phase angle α *z*-score was significantly lower compared to controls in both CD and UC patients as well as among boys and girls with IBD. Interestingly, although *z*-score in UC patients did not differ significantly from their healthy matches regarding height, weight, BMI and grip strength, phase angle α was significantly lower (*p* = 0.002) [25]. Our findings demonstrated significantly lower PhA in girls with UC (*P* = 0.038) and in boys with CD (*P* = 0.001). These differences were associated with lower body cell mass (*P* = 0.031 for girls with UC and *P* = 0.020 for boys with CD). A relatively high number of children and adolescents with PhA score below the 5th percentile in both UC and CD, and almost half of children below the 2nd percentile of FFMI score in patients with CD indicate high risk of malnutrition among children and adolescents hospitalized due to IBD. Also, BCM can be considered as a useful indicator of nutritional status in pediatric patients with IBD, due to its independency of hydration changes which occur with the disease [4]. 

Despite our best efforts and inclusion to our study, almost all pediatric patients with IBD from the Podkarpackie Region, one of the most important limitation of our study is relatively low number of study participants. It did not allow us to find other significant differences between study and control groups. Also, due to the fact that assessment of important markers of inflammation (ESR, CRP) and nutritional status (albumin and/or pre-albumin level) was not done in all the children, we were not able to analyze association of these variables with BIA and PhA results. Searching for such relationships would be an intriguing implication for further research.

According to our findings and ESPEN recommendations, we suggest that phase angle score should be used mandatorily by clinicians, especially in IBD children and adolescents hospitalized in pediatric gastroenterology units to identify children at high risk of malnutrition. The predictive and prognostic value of our results needs to be determined in further, long-term prospective studies to assess its role in the IBD population.

## 5. Conclusions

Lower phase angle score together with lower body composition parameters and selected nutrition indicators in IBD children and adolescents demonstrate their worse nutritional and functional status compared to healthy subjects. To assess the predictive and prognostic value of these findings, further prospective studies in this population are required.

## Figures and Tables

**Table 1:** Demographic and anthropometric parameters of the study participants.

Parameter	Ulcerative Colitis (UC) (Mean ± SD) (*n* = 34)	Control for UC (Mean ± SD) (*n* = 34)	*p*	Crohn’s Disease (CD) (Mean ± SD) (*n* = 25)	Control for CD (Mean ± SD) (*n* = 25)	*p*
Age (years)	13.5 ± 3.41	13.5 ± 3.41	1.000	13.8 ± 3.12	13.8 ± 3.12	1.000
Girls	13.1 ± 3.41	13.1 ± 3.41	1.000	13.1 ± 3.94	13.1 ± 3.94	1.000
Boys	13.9 ± 3.46	13.9 ± 3.46	1.000	14.1 ± 2.74	14.1 ± 2.74	1.000
Body weight (kg)	47.9 ± 15.33	49.2 ± 15.82	0.731	43.6 ± 14.14	54.9 ± 17.35	0.015
Girls	42.7 ± 13.25	45.1 ± 11.47	0.560	43.8 ± 15.99	51.0 ± 18.59	0.411
Boys	53.1 ± 15.85	53.3 ± 18.69	0.984	42.6 ± 13.72	56.8 ± 17.00	0.018
Height (cm)	156.7 ± 18.24	156.6 ± 17.68	0.981	155.9 ± 18.26	160.9 ± 17.64	0.316
Girls	150.8 ± 16.69	152.3 ± 14.73	0.783	148.4 ± 17.05	154.1 ± 20.84	0.551
Boys	162.6 ± 18.27	160.9 ± 19.71	0.795	159.4 ± 18.20	164.1 ± 15.58	0.411
BMI (kg/m^2^)	19.1 ± 3.55	19.6 ± 3.46	0.418	17.5 ± 3.41	20.6 ± 4.00	0.004 *
Girls	18.6 ± 3.98	19.2 ± 3.17	0.339	19.1 ± 4.14	20.7 ± 4.56	0.645
Boys	19.6 ± 3.11	20.0 ± 3.79	0.786	16.7 ± 2.82	20.6 ± 3.86	0.002 *
BMI *z*-score	−0.01 ± 0.928	0.13 ± 0.887	0.351	−0.42 ± 0.892	0.41 ± 1.050	0.003 *
Girls	−0.15 ± 1.045	0.05 ± 0.829	0.231	−0.03 ± 1.126	0.46 ± 1.211	0.505
Boys	0.14 ± 0.799	0.22 ± 0.959	0.812	−0.61 ± 0.725	0.38 ± 1.004	0.002 *
Disease activity, *n* (boys.girls) (PUCAI/PCDAI): Lack Mild Moderate Severe	12 (8/4) 8 (4/4) 10 (5/5) 4 (0/4)	n/a	n/a	7 (4/3) 5 (2/3) 9 (8/1) 4 (3/1)	n/a	n/a
Duration of the disease: New diagnosis Up to 1 year More than 1 year	16 4 14	n/a	n/a	10 3 12	n/a	n/a
Location of lesions (Paris classification):	27^P^ 3^L^ 4^E^	n/a	n/a	1^L1^ 2^L2^ 3^L3^ 1^L4b^ 2^L1/L4b^ 6^L1/L4a^ 10^L3/L4a^	n/a	n/a

SD—standard deviation; BMI—body mass index; *z*-score-standard score; PUCAI—Paediatric Ulcerative Colitis Activity Index; PCDAI—Paediatric Crohn’s Disease Activity Index; P—Pancolitis; ^L^—Left-sided; ^E^—Extensive; ^L1^—1/3 of distal section of ileum; ^L2^—The colon; ^L3^—The ileum and colon; ^L4b^—The upper section distally to the ligament of Treitz and proximally to 1/3 of the distal section of ileum; ^L1/L4b^—1/3 of the distal section of ileum and the upper section distally to the ligament of Treitz and proximally to 1/3 of the distal section of ileum; ^L1/L4a^—1/3 of the distal section of ileum and the upper section of the alimentary canal proximally to the ligament of Treitz; ^L3/L4a^—The ileum, colon and the upper section of alimentary canal proximally to the ligament of Treitz; n/a—not applicable. * indicate significant values (*p* < 0.05).

**Table 2:** Bioelectrical Impedance Analysis results in study participants.

Parameter	Ulcerative Colitis (UC) (Mean ± SD) (*n* = 34)		Control for UC (Mean ± SD) (*n* = 34)		*p*	Crohn’s Disease (CD) (Mean ± SD) (*n* = 25)		Control for CD (Mean ± SD) (*n* = 25)		*p*
FM (kg)	9.83	5.02	10.02	5.88	0.893	8.58	4.72	12.00	6.77	0.054
FM (%)	20.9	7.5	20.4	9.5	0.826	19.8	6.2	21.4	8.2	0.440
FMI	4.05	1.98	4.14	2.52	0.787	3.53	1.77	4.57	2.40	0.123
FFM (kg)	38.10	12.94	39.21	13.38	0.727	35.02	11.70	42.93	13.53	0.032 *
FFM (%)	79.1	7.5	79.6	9.5	0.826	80.2	6.2	78.6	8.2	0.440
FFMI	15.00	2.59	15.41	2.46	0.503	13.94	2.44	16.02	2.54	0.005 *
MM (kg)	24.24	9.86	26.15	10.01	0.432	21.80	8.39	28.39	9.90	0.015 *
MM (%)	49.5	8.2	52.4	7.9	0.137	49.1	5.9	51.5	7.2	0.199
TBW (liters)	29.51	8.90	30.24	9.31	0.741	27.86	7.69	32.97	9.37	0.040 *
TBW (%)	62.1	6.4	62.2	9.3	0.956	65.2	8.5	61.1	7.8	0.078
ECW (liters)	13.25	4.72	13.26	4.31	0.996	12.02	3.98	14.37	4.54	0.058
ECW (%)	44.7	6.7	43.8	3.4	0.552	43.0	5.4	43.4	3.0	0.779
ICW (liters)	16.24	5.05	16.98	5.18	0.345	15.83	4.11	18.60	5.02	0.038 *
ICW (%)	55.3	6.7	56.2	3.4	0.552	57.0	5.4	56.6	3.0	0.779
BCM (kg)	19.58	8.20	21.26	8.27	0.405	17.60	6.92	23.08	8.13	0.013 *
BCM (%)	49.4	8.8	53.3	3.9	0.066	49.3	5.3	53.1	3.2	0.004 *
BCMI	7.63	2.01	8.31	1.78	0.149	6.98	1.80	8.57	1.74	0.003 *
Resistance (ohm)	654.15	130.36	623.71	106.30	0.295	700.68	118.50	606.56	103.74	0.004 *
Reactance (ohm)	61.74	13.12	64.18	8.17	0.361	63.36	10.39	61.88	6.89	0.556
PhA	5.34	1.34	5.96	0.76	0.040*	5.16	1.18	5.90	0.62	0.009 *

SD—standard deviation; FM—fat mass; FMI—fat mass index; FFM—fat free mass; FFMI—fat free mass index; MM—muscle mass; TBW—total body water; ECW—extracellular water; ICW—intracellular water; BCM—body cell mass; BCMI—body cell mass index; PA—phase angle. * indicate significant values (*p* < 0.05).

**Table 3:** BIA results in UC participants according to gender.

Parameter	Girls (Mean ± SD)				*p*	Boys (Mean ± SD)				*p*
	Ulcerative Colitis (UC)		Control			Ulcerative Colitis (UC)		Control		
	(Mean ± SD) (*N* = 17)		(Mean ± SD) (*N* = 17)			(Mean ± SD) (*N* = 17)		(Mean ± SD) (*N* = 17)		
FM (kg)	9.98	5.79	11.25	4.57	0.274	9.69	4.28	8.79	6.87	0.193
FM (%)	23.0	8.1	24.6	7.2	0.838	18.7	6.5	16.2	9.9	0.160
FMI	4.45	2.40	4.85	2.17	0.496	3.66	1.40	3.42	2.70	0.218
FFM (kg)	32.68	9.86	33.89	8.64	0.518	43.52	13.63	44.54	15.31	0.786
FFM (%)	77.0	8.1	75.4	7.2	0.838	81.3	6.5	83.8	9.9	0.160
FFMI	14.08	2.19	14.31	1.84	0.563	15.91	2.68	16.50	2.56	0.474
MM (kg)	19.58	6.95	22.05	6.20	0.182	28.91	10.30	30.25	11.51	0.658
MM (%)	45.7	8.1	48.7	5.3	0.114	53.2	6.6	56.1	8.5	0.122
TBW (liters)	25.10	6.24	25.85	5.67	0.474	33.91	9.13	34.63	10.28	0.658
TBW (%)	59.9	6.2	58.1	7.1	0.474	64.4	6.1	66.3	9.7	0.290
ECW (liters)	11.68	4.70	11.29	2.79	0.865	14.82	4.30	15.23	4.73	0.658
ECW (%)	45.6	8.0	43.5	2.9	0.322	43.7	5.3	44.1	3.8	1.000
ICW (liters)	13.42	2.97	14.56	3.04	0.092	19.05	5.18	19.40	5.80	0.892
ICW (%)	54.4	8.0	56.5	2.9	0.322	56.3	5.3	55.9	3.8	1.000
BCM (kg)	15.69	5.79	17.92	5.11	0.170	23.48	8.54	24.59	9.55	0.658
BCM (%)	46.0	10.6	52.4	3.3	0.031 *	52.9	4.7	54.2	4.4	0.433
BCMI	6.77	1.76	7.55	1.25	0.114	8.49	1.92	9.06	1.94	0.339
Resistance (ohm)	700.12	110.30	685.18	79.06	0.433	608.18	135.63	562.24	95.00	0.322
Reactance (ohm)	61.59	14.35	68.94	8.19	0.140	61.88	12.20	59.41	4.74	0.786
PhA	5.06	1.23	5.76	0.63	0.038 *	5.61	1.43	6.15	0.85	0.290

BIA— Bioelectrical Impedance Analysis; UC—Ulcerative Colitis; SD—standard deviation; FM—fat mass; FMI—fat mass index; FFM—fat free mass; FFMI—fat free mass index; MM—muscle mass; TBW—total body water; ECW—extracellular water; ICW—intracellular water; BCM—body cell mass; BCMI—body cell mass index; PhA—phase angle. * indicate significant values (*p* < 0.05).

**Table 4:** BIA results in CD participants according to gender.

Parameter	Girls (Mean ± SD)				*p*	Boys (Mean ± SD)				*p*
	Crohn’s Disease (CD)		Control			Crohn’s Disease (CD)		Control		
	(Mean ± SD)		(Mean ± SD)			(Mean ± SD)		(Mean ± SD)		
	(*N* = 8)		(*N* = 8)			(*N* = 17)		(*N* = 17)		
FM (kg)	11.69	6.76	14.85	7.22	0.279	7.12	2.51	10.66	6.32	0.099
FM (%)	25.7	5.3	28.2	4.4	0.279	17.0	4.5	18.2	7.6	0.865
FMI	5.06	2.29	5.99	2.24	0.279	2.80	0.82	3.90	2.23	0.339
FFM (kg)	31.95	10.21	36.15	11.95	0.382	36.46	12.36	46.12	13.36	0.062
FFM (%)	74.3	5.3	71.8	4.4	0.279	83.0	4.5	81.8	7.6	0.865
FFMI	14.03	2.12	14.68	2.44	0.721	13.90	2.63	16.65	2.40	0.007 *
MM (kg)	20.63	7.31	22.74	7.69	0.505	22.36	9.02	31.05	9.89	0.022 *
MM (%)	47.3	5.7	44.9	3.2	0.382	50.0	5.9	54.6	6.4	0.045 *
TBW (liters)	24.51	6.84	27.01	7.98	0.382	29.43	7.74	35.78	8.82	0.038 *
TBW (%)	57.9	7.0	54.4	6.1	0.234	68.7	6.8	64.2	6.4	0.099
ECW (liters)	10.51	2.92	11.90	3.73	0.382	12.74	4.29	15.54	4.51	0.106
ECW (%)	43.6	5.2	44.1	2.9	0.328	42.8	5.6	43.0	3.1	0.892
ICW (liters)	14.00	4.12	15.11	4.36	0.328	16.69	3.93	20.24	4.54	0.012 *
ICW (%)	56.5	5.2	55.9	2.9	0.328	57.2	5.6	57.0	3.1	0.892
BCM (kg)	16.76	6.07	18.44	6.28	0.645	17.99	7.44	25.26	8.13	0.020 *
BCM (%)	51.5	5.2	50.7	2.4	0.505	48.3	5.2	54.2	2.9	<0.001 *
BCMI	7.30	1.51	7.46	1.39	0.959	6.82	1.94	9.09	1.68	0.003 *
Resistance (ohm)	711.13	82.97	682.75	92.97	0.574	695.76	134.06	570.71	89.97	0.004 *
Reactance (ohm)	69.75	11.63	64.75	8.31	0.645	60.35	8.53	60.53	5.91	0.865
PhA	5.65	0.92	5.44	0.44	0.442	4.94	1.24	6.12	0.59	0.001 *

SD—standard deviation; FM—fat mass; FMI—fat mass index; FFM—fat free mass; FFMI—fat free mass index; MM—muscle mass; TBW—total body water; ECW—extracellular water; ICW—intracellular water; BCM—body cell mass; BCMI—body cell mass index; PhA—phase angle. * indicate significant values (*p* < 0.05).

**Table 5:** BIA results in UC vs. CD participants according to percentiles.

Parameter	Ulcerative Colitis (UC) (*n* = 34)		Control for UC (*n* = 34)		*p*	Crohn’s Disease (CD) (*n* = 25)		Control for CD (*n* = 25)		*p*
	N	%	N	%		N	%	N	%	
FM (kg) > 9.P *	32	94.1	30	88.2		25	100.0	24	96.0	
FM (kg) < 9.P *	-	-	1	2.9	0.531	-	-	-	-	0.312
FM (kg) < 2.P *	2	5.9	3	8.8		-	-	1	4.0	
FFM (kg) > 9.P *	34	100.0	34	100.0		24	96.0	24	96	
FFM (kg) < 9.P *	-	-	-	-	-	1	4.0	1	4.0	0.755
FFM (kg) < 2.P *	-	-	-	-		-	-	-	-	
FMI > 9.P **	23	67.6	26	76.5		20	80.0	22	88.0	
FMI < 9.P **	8	23.5	4	11.8	0.436	4	16.0	3	12.0	0.538
FMI < 2.P **	3	8.8	4	11.8		1	4.0	-	-	
FFMI > 9.P **	25	73.5	30	88.2		12	48.0	22	88.0	
FFMI < 9.P **	4	11.8	3	8.8	0.196	2	8.0	1	4.0	0.009 ^†^
FFMI < 2.P **	5	14.7	1	2.9		11	44.0	2	8.0	
PhA > 10.P ***	26	76.5	20	58.8		18	72.0	24	96.0	
PhA < 10.P ***	1	2.9	13	38.2	<0.001 ^†^	-	-	1	4.0	0.012 ^†^
PhA < 5.P ***	7	20.6	1	2.9		7	28.0	-	-	

FM—fat mass; FMI—fat mass index; FFM—fat free mass; FFMI—fat free mass index; PhA—phase angle; P—percentile; * According to Wells et al. reference values [27]; ** According to Wells et al. reference values [28]; *** According to Bosy-Westhpal et al. reference values [20]. ^†^ indicate significant values (*p* < 0.05).
